# Novel Insight Into Nutritional Regulation in Enhancement of Immune Status and Mediation of Inflammation Dynamics Integrated Study *In Vivo* and *In Vitro* of Teleost Grass Carp (*Ctenopharyngodon idella*): Administration of Threonine

**DOI:** 10.3389/fimmu.2022.770969

**Published:** 2022-03-14

**Authors:** Yu-Wen Dong, Wei-Dan Jiang, Pei Wu, Yang Liu, Sheng-Yao Kuang, Ling Tang, Wu-Neng Tang, Xiao-Qiu Zhou, Lin Feng

**Affiliations:** ^1^ Animal Nutrition Institute, Sichuan Agricultural University, Chengdu, China; ^2^ Fish Nutrition and Safety Production University Key Laboratory of Sichuan Province, Sichuan Agricultural University, Chengdu, China; ^3^ Key Laboratory for Animal Disease-Resistance Nutrition of China Ministry of Education, Sichuan Agricultural University, Chengdu, China; ^4^ Animal Nutrition Institute, Sichuan Academy of Animal Science, Chengdu, China

**Keywords:** threonine, immune status, inflammation, *in vivo* and *in vitro*, juvenile grass carp

## Abstract

This study aims to investigate the effects of threonine (Thr) on immunoregulation *in vivo* and *in vitro* of teleost grass carp (*Ctenopharyngodon idella*). Juveniles (9.53 ± 0.02 g) were reared for 8 weeks with respective Thr diet (3.99, 7.70, 10.72, 14.10, 17.96, and 21.66 g/kg) and then challenged with *Aeromonas hydrophila* for *in vivo* study. Macrophages isolated from head kidney were treated *in vitro* for 48 h with L-Thr (0, 0.5, 1.0, 2.0, 4.0, and 8.0 mM) after 6 h of lipopolysaccharide induction. The results showed that, compared with Thr deficiency (3.99 g/kg), the optimal dietary Thr (14.10g/kg) affected the immunocyte activation in the head kidney (HK) and spleen (SP) by downregulating the mRNA expressions of MHC-II and upregulating CD4 (not CD8), and it mediated the innate immune by enhancing the activities of lysozyme (LZ), acid phosphatase content of complement 3 (C3) and C4, increasing the mRNA abundances of hepcidin, liver expressed antimicrobial peptide-2A (LEAP-2A), LEAP-2B, β-defensin1, downregulating tumor necrosis factor α (TNF-α), IL-6, IL-1β, IL-12p35, IL-12p40, IL-17AF1, and IL-17D partly by attenuating RORγ1 transcriptional factor and nuclear factor kappa B p65 (NF-κBp65) signaling cascades [IKKβ/IκBα/NF-κBp65] and upregulating transforming growth factor β1 (TGF-β1), IL-4/13A, -4/13B, IL-10, and IL-22 partly by GATA-3. Besides these, the optimal dietary Thr regulated the adaptive immune by upregulating the mRNAs of immunoglobulin M (IgM) and IgZ (not IgD). Moreover, 2 mM Thr downregulated *in vitro* the mRNA abundances of colony stimulating factor-1, inducible nitric oxide synthase, mannose receptor 1, matrix metalloproteinase2 (MMP-2), and MMP-9 significantly (*P* < 0.05), indicating that Thr could attenuate the M1-type macrophages’ activation. Moreover, L-Thr downregulated the mRNA transcripts of TNF-α, IL-6, and IL-1β associated with impairing the SOCS1/STAT1 signaling and upregulated IL-10 and TGF-β1 partly by accentuating the SOCS3/STAT3 pathway. The above-mentioned observations suggested that Thr improved the immune status in the immune organs of fish by enhancing the immune defense and mediating the inflammation process. Finally, based on the immune indices of LZ activity in HK and C3 content in SP, the optimal Thr for immune enhancement in juvenile grass carp (9.53–53.43 g) was determined to be 15.70 g/kg diet (4.85 g/100 g protein) and 14.49 g/kg diet (4.47 g/100 g protein), respectively.

## Introduction

Nutrition-immune have been a focal point with the aim of improving animal health (1, 2). Numerous studies demonstrated that nutrient supplements not only promoted growth but also enhanced disease resistance, which were highly dependent on the immune system to trigger an effective defense response against the pathogen (3–8). Not like the mammals, teleosts have developed multiple and sophisticated immune organs, including the gill, intestine, skin, head kidney, spleen, and so on (9–11). Nutrient-targeted immunoregulation was mainly focused on the intestine response locally, which depended on tissue-resident immunocyte activation and, necessarily, the renewal or replenishment macrophages derived from lymph nodes or bone marrow upon suffering from antigen attacks (12–16). Teleosts have a reserved hematopoietic function in the spleen but lack mature lymphatic organs with a substitution of the head kidney, thus being considered as the key core that coordinated immunoregulation as well as emphasized with highly expressed clusters of immune enzymes and metabolism- and transcriptional factor-related genes (17–20). A mammalian study demonstrated that an immune reaction is initiated by, but not limited to, the antigen-stimulated activation of antigen-presenting cells (APCs), antimicrobial substances involved defenses, cytokines that mediated the migration of macrophages, and inflammatory regulated T lymphocytes that processed immunological balances (21–23). As the essential nutrients’ affordance in fish, amino acids play a vital role in energy consumption and metabolic alteration under normal or abnormal conditions (24). Recently, a comparison of tryptophan and methionine target regulating the immune in the head kidney and blood was well documented. It was clearly shown that inflammation suppressed by the effects of tryptophan than methionine, with a notable downregulation in mmp9 and IL-1β of juvenile European seabass (25). Arginine was confirmed to have no remarkable alteration for the inflammatory process but in IL-10 and IL-34 regulation in the head kidney of juvenile gilthead seabream (26). Thus, different effects addressed on immunoregulation by amino acids might be diverse in fish. To date, evidence focused on the systematical administration of amino acids on teleost’s immune regulation from immune cell activation, innate immune component production to inflammation network-mediated signaling cascade crosstalk as well as the underlying mechanism in the head kidney (HK) and spleen (SP) of fish, the data of which are still limited.

Threonine (Thr) has been proven to be an indispensable amino acid for the optimal growth of an animal and functionally involved in many physiological and biochemical processes, including incorporating into mucins (27) and immunoglobulins (28), stimulating lymphocyte proliferation (29), and degrading ketone metabolites (propionate and butyrate) (30). As the immune response supporters, APCs, inflammatory stimulated macrophages, antibody-produced B lymphocytes, cytokine-regulated T lymphocytes, and phagocytes co-contributed to the immunomodulation wherever they localized in tissues or organs (31–35). With regards to initializing the immune process, APC activation takes place along with the highly expressed major histocompatibility complex (MHC) in cell surface, subsequently resulting in macrophage stimulation with highly upregulated colony-stimulating factor 1 (CSF1) and mannose receptors (MRCs) (36–38). Meanwhile, CD4- and CD8-expressed T lymphocytes could protect the immune reaction from disorders (39). To date, there has been no research regarding the effects of Thr on immunocyte activation in fish. Being Thr-derived metabolites, butyrate could downregulate the MHC-II gene abundance in the distal intestine of juvenile hybrid grouper (40), and the membrane-bond mucin could drive the formation of TAM macrophages derived from monocytes in human (41). Hamard et al. (42) have confirmed that dietary Thr supplementation could upregulate the B-cell translocation gene 1 protein (BTG1) gene expression in the ileum of piglets (42). In mice, the depletion of BTG1 could decrease the numbers and population of naïve CD4^+^ and CD8^+^ T cells in the periphery (43) and also defect the progenitor B-cell differentiation in the spleen (44). Hence, the possibility exists that Thr could regulate the immune progress by the contribution of immunocyte activation in fish, which deserves investigation. Besides this, immune modulation integrated the defense response by phagocytes releasing lysozyme and defensins, macrophage producing cytokines, and the minimizing immune overreaction by T cell-derived interleukins (45). In spite of previous studies in animal intestines that have documented the detailed alternation of dietary Thr on the activities of lysozyme, contents of complements, and cytokines expression (46–49), incompatible results still occurred and could not be generalized as a whole. Furthermore, an *in vitro* study addressed the Thr targeting the immunoregulation as well as involved signaling mechanism that remains unclear. As the canonical pathway, nuclear factor-kappa B (NF-κB) is pointed out as the key signaling cascade enlarging the inflammatory cytokine expression in human (50). In SKOV3 cells, cytokines, like IL-6, could feedback the inflammation to the signal transducer and activator of transcription (STAT) family for adjusting the immune progress (51). However, no study demonstrated the signaling regulation by which Thr might mediate the immunocyte activation in an *in vitro* animal study. Hamard et al. (52) reported that dietary Thr supplementation increased the glutamine levels in the plasma of early-weaned pigs. It was confirmed that glutamine could inhibit NF-κB expression in the liver of rats (53). In mouse embryonic stem cells, a research concluded the requirement of Thr for the synthesis of S-adenosylmethionine (SAM) (54). As the principal methyl contributor, SAM could magnify the JAK/STAT3 signaling when acting as anti-tumor in human pancreatic cancer (55). The above-mentioned observations implicated that Thr might work on the interaction of NF-κB- and STAT-related signaling cascades *in vivo* and *in vitro*, which is worthy of further exploration.

Therefore, to better understand the role of Thr in immunological regulation, grass carp (*Ctenopharyngodon idella*) was used as teleost model, and the head kidney (HK) and spleen (SP) were targeted as the main organs with macrophages in HK for *in vitro* verification. Particularly, attention was drawn to antibacterial compounds, immunocyte-activated biomarkers, multiple isoforms of immunoglobulin response, pro- and anti-inflammation as well as NF-κB and JAK/STAT signaling dynamics to heighten the comprehension of immunomodulation by administration of Thr.

## Materials and Methods

### Ethics Statement

According to the approved guidance standards by the National Institutes of Health during the operation for experimental animals (NIH Publication No. 8023), all the experiment protocols, especially the precautions for animal handling, were followed carefully and permitted by the Animal Care Advisory Committee in Sichuan Agricultural University (grant no. B20151714).

### Diets and Fish Husbandry

The diet formulation and nutrient composition are displayed in **Supplementary Table S1** (56). Dietary essential amino acids were profiled with whole-body amino acid patterns of grass carp, excluding Thr as referred to by Wang et al. (57). According to Tang et al. (58), an iso-nitrogenous diet was prepared by supplementing with glycine instead of incremental Thr. After producing the mixture, L-Thr in each diet was determined as 3.99, 7.70, 10.72, 14.10, 17.96, and 21.66 g/kg, respectively.

All the experiment protocols, especially for the precautions of animal handling, were designed and permitted by the Animal Care Advisory Committee in Sichuan Agricultural University (grant no. B20151714). Before starting the feeding trial, the juveniles were kept acclimatized in an experimental environment for 4 weeks after having been transported from a fish farm. Then, 1,080 juveniles (initial weight, 9.53 ± 0.02 g) were randomly distributed to 18 net cages, with an average of 60 juveniles in each cage. A disc (100 cm diameter) was set at the bottom of each cage for collection of uneaten feeds, with reference to the reported method in our lab (59). Feeding frequency was adopted as four times in a day, and the water temperature and pH value were regularly determined as 28 ± 2°C and 7.0 ± 0.5, respectively. The dissolved oxygen in the water was not less than 6.0 mg/L. All the experimental treatments were performed under natural 12-h light and dark cycle, respectively.

### *A. hydrophila* Challenge Test *In Vivo*

*Aeromonas hydrophila*, a kind of heterotrophic, Gram-negative bacterium, was commonly spreading as an emerging pathogenic bacterium which caused great loss in aquaculture production and had a confirmed multiple pathogenicity with potential amounts of extracellular proteins like aerolysin, lipase, chitinase, and enterotoxins, thus being usually used as challenge test bacterium (60). After the growth trial, the successfully established disease-resistance test was used as described in our previous study (56). Briefly, sixty juveniles from each dietary Thr treatment group were selected randomly and then placed in another labeled empty cage respectively for acclimatization for 5 days. Meanwhile, *A. hydrophila* was cultured in lauryl tryptose medium (peptone 10.0 g/L, yeast extract 15.0 g/L, sucrose 7.0 g/L, and K_2_HP0_4_ 4.56 g/L) at 28°C with shaking incubation at 180 rpm/min. Then, 1.0 ml *A. hydrophila* was injected into the intraperitoneal cavity of juveniles with a dosage of 2.5 × 10^5^ colony-forming units (cfu)/ml. This injection concentration was determined to be of a nonlethal dosage that could induce inflammation response according to our preliminary test. The challenge trial was conducted for 14 days, and the feeding conditions agreed with those in the growth experiment.

At the ending of the challenge trial, all juveniles from each dietary Thr treatment group were anaesthetized by a benzocaine bath following Geraylou et al. (61). Quickly, the HK and SP organs were isolated, collected, labeled, and frozen in liquid nitrogen and then stored at -80°C for later use following the method of Chen et al. (62).

### Isolation and Primary Cultivation of Grass Carp Head Kidney Macrophages

Macrophages in the HK of grass carp were isolated according to the method of Meng et al. (63). Briefly, head kidney from a carp weighing 200 g was taken out carefully into the RPMI 1640 medium (Cat# 11875093, Gibco™) containing 2% fetal bovine serum (FBS; cat# A4766801, Gibco™) with 10 U/ml heparin (cat# H3149, Sigma-Aldrich), and 100 U/ml penicillin and streptomycin (cat# 15070063, Gibco™), washed three times, and then passed through a 200-gauge stainless mesh to a 15-ml tube. The cells were suspended by 5 ml Ficoll-Hypaque (1.08 kg/L, TBDscience, Tianjin, China) and then centrifuged at a speed of 500 g for 30 min at room temperature. The cells located in the upper liquid layer were extracted and removed to another 15-ml tube, then washed with 5 ml RPMI 1640 medium, and centrifuged at 500 g for 2 min, with the supernatants discarded. The cell pellets were resuspending with 1 ml RPMI 1640 medium containing 2% FBS and 100 U/ml penicillin and streptomycin. The cells were counted using trypan blue dye to adjust the number at 1 × 10^6^ per well, seeded in 24-well plates (Nunc), and then incubated at 27°C with 5% carbon dioxide for 2 h. After that, the non-adherent cells were gently discarded, and the culture medium was replenished with RPMI 1640 containing 2% FBS and 100 U/ml penicillin and streptomycin for cultivation at 12 h.

Prior to the treatment by L-Thr (Cat# 72-19-5, Sigma), the non-adherent cells were removed and then washed by sterile PBS. Then, the cells were treated with RPMI 1640 medium containing lipopolysaccharide (LPS) (40 ug/ml, Sigma) for 6 h. After that, the customized RPMI 1640 medium containing 100 U/ml penicillin and streptomycin was supplemented by respective levels of L-Thr (0, 0.5, 1.0, 2.0, 4.0, and 8.0 mM) and then incubated for 48 h. Head kidney macrophages were collected after lysing with 100 μl 0.1% Triton X-100 (cat# HFH10, Themo Fisher) at 4°C for 30 min and stored at -80°C in a freezer for later analysis subsequently.

### Histological Observation

Tissue samples of head kidney and spleen were washed carefully for three times and fixed with 4% paraformaldehyde and then dehydrated by a graded dosage of ethanol. Traditional methods of tissue paraffin embedding, sectioning, and hematoxylin and eosin staining (H&E staining) were conducted according to the method of Reyes-Becerril et al. (64). A light microscope (Nikon TS100, Japan) was used to examine the histological characteristics, which was determined by the software Image-Pro^®^ Plus v 6.0.

### Biochemical Parameter Analysis

Tissue homogenates of HK and SP were prepared by dilution of 10 times the volume (w/v) of ice-cold normal saline. Then, tissues were cut into small pieces quickly by scissors and centrifuged at 4°C, 6,000 *g* for 20 min. The supernatants were gently removed and stored for determining immune-related enzymes according to the method of Pan et al. (17). The activities of lysozyme and acid phosphatase (ACP) and the contents of complement 3 (C3) and C4 were measured by commercial kits (Nanjing Jiancheng Bioengineering Institute, China), as reported in the study of Zhang et al. (65)

### Real-Time Polymerase Chain Reaction Analysis

RNA extraction, reverse transcription, and quantitative real-time PCR for target genes were conducted according to the reported method from our laboratory (66). Briefly, total RNA was extracted from HK, SP, and cell samples using 1 ml RNAiso Plus kits (cat# 9109, TaKaRa, Liaoning, China) under the reagent manufacturer’s instructions. Then, the quality of the isolated RNA was evaluated by 1% agarose gel electrophoresis analysis and the quantity by the definition with s spectrophotometry (Nanodrop 2000, Themo Fisher Scientific Inc., USA), respectively. Finally, using the Prime Script™ RT Reagent Kit (cat# RR047A, TaKaRa), RNA was transcribed reversely to cDNA according to the manufacturer’s instruction. For quantitative real-time PCR, specific primers were designed by referring to the cloned sequences in our lab and those released from the NCBI website (**Table 1**). As preliminary test regarding the evaluation for internal control genes (data not shown), β-actin was used as a reference gene to normalize cDNA loading. Amplificated efficiency in each primer of the target genes was calculated as approximately 100% by referring to the standard curves of a specific gene generated from 10-fold serial dilutions. The qPCR thermal reaction volume was 15 μl, containing 2 μl cDNA, 7.5 μl SYBR^®^ Green dye (BioRad, USA), 0.5 μl (10 μM) of each primer, and 4.5 μl PCR-grade water. Simplified operating procedures were observed, starting with pre-heating for 5 min at 95°C, one cycle running at 95°C for 5 s and 60°C (annealing temperature) for 30 s, and then followed with 40 cycles. Melt curves were analyzed to verify the single peak for specific primers. Relative quantification was adopted by the 2^−ΔΔCT^ calculation formula to normalize the target gene expression according to the method of Livak and Schmittgen (67).

**Table 1:** Real-time PCR primer sequences.

Target gene	Full Name	Forward primer (5’→3’) Reverse primer (5’→3’)	Temperature (°C)	Accession no.
Hepcidin	Hepcidin	AGCAGGAGCAGGATGAGC GCCAGGGGATTTGTTTGT	59.3	JQ246442.1
LEAP-2A	Liver expressed antimicrobial peptide 2A	TGCCTACTGCCAGAACCA AATCGGTTGGCTGTAGGA	59.3	FJ390414
LEAP-2B	Liver expressed antimicrobial peptide 2B	TGTGCCATTAGCGACTTCTGAG	59.3	KT625603
		ATGATTCGCCACAAAGGGG		
β-Defensin1	Beta defensin 1	TTGCTTGTCCTTGCCGTCT	58.4	KT445868
		AATCCTTTGCCACAGCCTAA		
IFN-γ2	Interferon gamma 2	TGTTTGATGACTTTGGGATG	60.4	JX657682
		TCAGGACCCGCAGGAAGAC		
TNF-α	Tumor necrosis factor α	CGCTGCTGTCTGCTTCAC	58.4	HQ696609
		CCTGGTCCTGGTTCACTC		
IL-1β	Interleukin-1 β	AGAGTTTGGTGAAGAAGAGG	57.1	JQ692172
		TTATTGTGGTTACGCTGGA		
IL-4/13A	Interleukin-4/13 A	CTACTGCTCGCTTTCGCTGT	55.9	KT445871
		CCCAGTTTTCAGTTCTCTCAGG		
IL-4/13B	Interleukin-4/13 B	TGTGAACCAGACCCTACATAACC	55.9	KT625600
		TTCAGGACCTTTGCTGCTTG		
IL-6	Interleukin-6	CAGCAGAATGGGGGAGTTATC	62.3	KC535507.1
		CTCGCAGAGTCTTGACATCCTT		
IL-10	Interleukin-10	AATCCCTTTGATTTTGCC	61.4	HQ388294
		GTGCCTTATCCTACAGTATGTG		
IL-12p35	Interleukin-12 p35	TGGAAAAGGAGGGGAAGATG	55.4	KF944667.1
		AGACGGACGCTGTGTGAGTGTA		
IL-12p40	Interleukin-12 p40	ACAAAGATGAAAAACTGGAGGC	59.0	KF944668.1
		GTGTGTGGTTTAGGTAGGAGCC		
TGF-β1	Transforming growth factor β 1	TTGGGACTTGTGCTCTAT	55.9	EU099588
		AGTTCTGCTGGGATGTTT		
TGF-β2	Transforming growth factor β 2	TACATTGACAGCAAGGTGGTG	55.9	KM279716
		TCTTGTTGGGGATGATGTAGTT		
CSF-1	Colony stimulating factor 1	CAGGTCTGAGCATCCTTGTTGAA	59.8	MK548356.1
		TTCCTGTCCTCCTGGGATTTG		
MRC1	Mannose receptor C-type 1	TCATCTCTTGTCAGCATTAGGGA	60.5	KF569903.1
		GTAATCTACCACGCTGTTGTCTGA		
iNOS	Inducible nitric oxide synthase	GTATCATGACAGCTTACTCTAGG	60.5	HQ589354.1
		GACTAAGATTTCCTGGATAGTGG		
CD4	Cluster of differentiation 4	ATACCTCTCTCTCCTCATCCTA	59.3	GQ355588.1
		AACCTGCTATCTTGACCCG		
CD8α	Cluster of differentiation 8 α	TCTTCTTCGGAGGGCTGACT	59.8	GQ355586.1
		CACAGTTGAAACGGGGCTT		
CD8β	Cluster of differentiation 8 β	AACAGTCAAGACCCAGAAACCT	59.3	GQ355587.1
		GCCAAAAGCAAAAGAGAACCAAT		
MHCIIα	Major histocompatibility complex class II α	TCTATGCCAGGAATGATGTG	60.0	EF140725.1
		ATTTGGGCGATACTGACTTA		
MHCIIβ	Major histocompatibility complex class II β	TGTCTGTATTCACTGGAGCAACTAA	60.0	EF140726.1
		CAAACTCTGTAAACCCCACAAATA		
SOCS1	Suppressor of cytokine signaling 1	ACCCTTCAACTATGCGAGAGACT	59.8	GU224284.1
		GTGTTGTCCTTTGTAACTCAGCG		
SOCS3	Suppressor of cytokine signaling 3	CCCATAACCATTCCCAGCA	59.3	EU625352.1
		TTGTAACGGTGAGACGGCAG		
GATA3	GATA binding protein 3	GTGGGCATCAAGGCACAAACAT	62.5	JX021295.1
		TCTCACGGGGGCAAAGAATAGG		
NF-κB p65	Nuclear factor kappa B p65	GAAGAAGGATGTGGGAGATG	62.3	KJ526214
		TGTTGTCGTAGATGGGCTGAG		
IκBα	Inhibitor of κB α	TCTTGCCATTATTCACGAGG	62.3	KJ125069
		TGTTACCACAGTCATCCACCA		
IKKα	IκB kinase α	GGCTACGCCAAAGACCTG	60.3	KM279718
		CGGACCTCGCCATTCATA		
IKKβ	IκB kinase β	GTGGCGGTGGATTATTGG	60.3	KP125491
		GCACGGGTTGCCAGTTTG		
IKKγ	IκB kinase γ	AGAGGCTCGTCATAGTGG	58.4	KM079079
		CTGTGATTGGCTTGCTTT		
IgD	Immunoglobulin D	TTGGTTGTTGGTCAGAGTGTCA	58.6	GQ429174.1
		TGGTTTTTGAGATGTTGTGCTG		
IgM	Immunoglobulin M	AGTCAATCTTCGCCCTGTCTCA	60.3	DQ417927.1
		TGGTGGAGTTGGAGGTAGACGA		
IgZ	Immunoglobulin Z	GACGGACACATCAAGCAGGAAAT	58.6	GQ201421.1
		CTCTTCGTAAGGCTTTTCTCTCA		
IL-15	Interleukin-15	CCTTCCAACAATCTCGCTTC	61.4	KT445872
		AACACATCTTCCAGTTCTCCTT		
IL-17AF1	Interleukin-17AF1	GGCATAGGACTGACAGAAGACA	58.6	KC978892.1
		ATGGAGAGATGGAGTCGTTGTT		
IL-17D	Interleukin-17D	GTGTCCAGGAGAGCACCAAG	62.3	KF245426.1
		GCGAGAGGCTGAGGAAGTTT		
IL-22	Interleukin-22	GTTCCTAACTCTGCTTGCTGTG	57.0	MN643172.1
		ACATAGAGGTTGTTCCAGGTGA		
RORγ1	RAR-related orphan receptor gamma 1	CCTGGATGACATAACCACGCTA	60.2	JN882281.1
		GAGAACTTTGGGAGGACGAACT		
RORγ2	RAR-related orphan receptor gamma 2	TTGGCTAAAAGTCTGAGTCGT	54.5	JN882282.1
		GGTAGTTTGGACACCATCTTG		
MMP-2	Matrix metalloproteinase 2	GCTGTGGACATTAGGAGAAGGC	59.8	HQ153830.1
		TCATCCCTTCCTGAGGTGGT		
MMP-9	Matrix metalloproteinase 9	CATAGGCACATGAAACGGGATG	58.3	HQ153831.1
		TCAACACAGAGAATGGATGCTT		
β-Actin	Beta-actin	GGCTGTGCTGTCCCTGTA	61.4	M25013
		GGGCATAACCCTCGTAGAT		

### Western Blotting Analysis

Protein samples from tissues or cells were prepared by RIPA lysis kit (cat# P0013B, Beyotime Biotechnology Inc., China), and the concentrations were determined by the BCA assay kit (P0012S, Beyotime Biotechnology Inc., China) according to the manufacturers’ instruction. Then, equivalent protein samples were loaded for SDS-PAGE electrophoresis, and subsequently the separated protein was moved into polyvinylidene fluoride membrane (PVDF) for blotting test. After blocking with bovine serum albumin (5%), the PVDF was dipped in diluted primary antibody solution for incubation at 4°C overnight. Lamin B1 and β-actin were the control proteins for total protein according to the procedure conducted in our lab (68, 69). Anti-NF-κBp65 (cat# DF7003), STAT1 (cat# AF6300), phospho-STAT1 (Tyr701) (cat# AF3300), STAT3 (cat# AF6300), and phospho-STAT3 (Tyr705) (cat# AF3293) were purchased from Affinity Bioscience Co., Ltd. After the primary antibody was incubated and subsequently washed with TBST, goat anti-rabbit IgG-HRP (cat# sc-2004, Santa Cruz Biotechnology, USA) was applied for secondary antibody incubation for 2 h at room temperature. Then, conjugation was visualized by using BeyoECL Star reagents (cat# P0018AS, Beyotime Biotechnology Inc., China) by ChemiDoc™ Touch Gel Image System (Bio-Rad, USA). The western bands were quantified by Image J software (version 1.63, NIH). The relative expression unit in each treatment was expressed as relative to the control group.

### Data and Statistical Analysis

All data were expressed as the mean value plus standard deviations. The homogeneity variance of the data in the *in vivo* study was verified before subjecting such to one-way analysis of variance (ANOVA), and the Duncan’s multiple-range tests were analyzed by SPSS statistic software (version 20.0, SPSS Inc., USA) to determine the difference among the treatments at the significance level of *P <*0.05 according to Jiang et al. (70). Two-tailed Student’s *t*-test was applied for data analysis in the *in vitro* study with significance level at *P <*0.05, 0.01, and 0.001 when compared among different groups, respectively.

## Result

### Histopathological Alteration in the HK and SP of Juveniles

**Figure 1:**
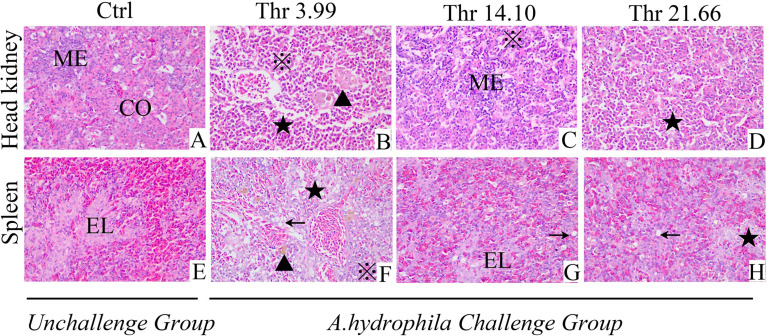
Histopathological changes under *Aeromonas hydrophila* challenge in the head kidney **(A–D)** and spleen **(E–H)** of juvenile grass carp (*Ctenopharyngodon idella*) fed diets containing graded levels of Thr for 8 weeks. H&E, ×400. Ctrl, unchallenged group supplemented with Thr 14.10 g/kg diet. Melanin macrophages center (▲), ellipsoid (EL), cortex (CO), medulla (ME), necrosis and dissolution of parenchymal cells (★); vacuolar degeneration (←), homogeneous red-stained inflammatory exudate, inflammatory cell infiltration (※).

Data on the histological examination is shown in **Figure 1**. As presented, compared with the optimal dietary Thr (14.10 g/kg) group with saline injection, the *A. hydrophila*-challenged group displayed much more melanin macrophage centers in the HK and ellipsoid-like vacuoles in the SP. Mover, after the *A. hydrophila* challenge, comparing with 14.10 g/kg Thr diet, juveniles fed the Thr 3.99-g/kg diet showed the worse tissue lesion with more hyperemia, inflammation cell infiltration in the HK, disintegrated splenic parenchymal cells (including lymphocytes and reticular cells), and decreased necrosis in the SP. Besides this, juveniles fed with Thr 21.66-g/kg diets showed fewer of the above-mentioned symptoms compared with the Thr-insufficient group (3.99 g/kg).

### Gene Expression of Immunocyte Activation Biomarkers in the HK and SP of Juveniles *In Vivo*

**Figure 2:**
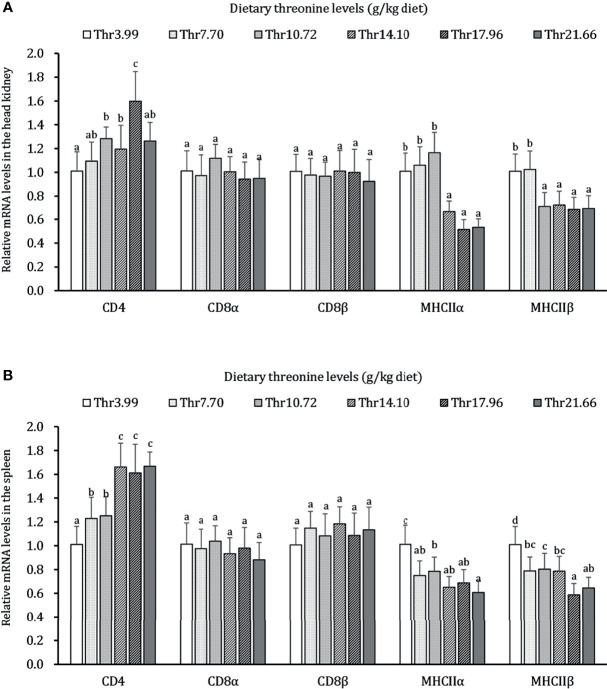
Relative mRNA expression of immunocyte activators in the head kidney **(A)** and spleen **(B)** of juvenile grass carp (*Ctenopharyngodon idella*) fed diets containing graded levels of Thr for 8 weeks. CD4, cluster of differentiation 4; CD8α, cluster of differentiation 8α; CD8β, cluster of differentiation 8β; MHCIIα, major histocompatibility complex class II α; MHCIIβ, major histocompatibility complex class II β. Data represent the means of six fish in each group. The error bars indicate SD. Values having different letters are significantly different (*P* < 0.05).

The effects of dietary Thr on immunocyte activation in the HK and SP of juveniles *in vivo* is presented in **Figure 2**. In the HK, CD4 mRNA abundance was upregulated gradually, with the dietary Thr increasing and reaching the peak (*P* < 0.05) when it was supplemented with 17.96 g/kg diet and then downregulated. The MHC-IIα and MHC-IIβ mRNA levels were maintained constantly (*P* > 0.05) and downregulated until the dietary Thr increased to 14.10 and 10.72-g/kg diet, respectively, and plateaued thereafter (*P* > 0.05). The dietary Thr did not affect the mRNA transcript abundances of CD8α and CD8β (*P* > 0.05). In the SP, CD4 mRNA expression was upregulated significantly (*P* < 0.05) as dietary Thr supplementation and achieved the highest level when it increased up to 14.10 g/kg diet and plateaued thereafter (*P* > 0.05). The mRNA expressions of MHC-IIα and MHC-IIβ were present the gradual decrease with dietary Thr supplementation. The mRNA levels of CD8α and CD8β did not show remarkable changes as response to dietary Thr supplementation(*P* > 0.05).

### Non-Specific Immune Active Substances Production in the HK and SP of Juveniles *In Vivo*

The effects of different levels of Thr diets on the activities of non-specific immune active substances lysozyme (LZ), ACP, and contents of C3 and C4 in the HK and SP of juveniles *in vivo* are shown in **Table 2**. In the HK, with dietary Thr addition of 14.10 g/kg, dramatic enhancements of LZ activity and C4 level were observed (*P* < 0.05), which gradually declined thereafter. The ACP activity and C4 content accumulated significantly (*P* < 0.05) as the supplemented dietary Thr was increased up to 10.72 g/kg diet, and all plateaued thereafter (*P* > 0.05). In the SP, with the incremental dietary Thr, the activities of ACP and LZ were generated significantly (*P* < 0.05) and achieved a maximum when the dietary Thr level reached 14.10 g/kg diet, thereafter remaining unchanged with a further increase of dietary Thr. The C3 content was enhanced as the incremental Thr was supplemented in the diet, while the highest level was observed in juveniles fed dietary Thr at 14.10 g/kg, and then it dropped off. Comparing with dietary Thr levels above 7.70 g/kg, Thr deficiency reduced the C4 contents significantly (*P* < 0.05).

**Table 2:** Non-specific immune enzyme parameters in the head kidney and spleen of juvenile grass carp (*Ctenopharyngodon idella*) fed diets containing graded levels of Thr for 8 weeks.

		Dietary threonine levels (g/kg)					
		3.99	7.70	10.72	14.10	17.96	21.66
Head kidney							
LZ^a^		142.83 ± 12.78^a^	157.48 ± 7.10^b^	175.95 ± 7.94^c^	201.99 ± 5.15^d^	185.36 ± 15.53^c^	182.37 ± 11.60^c^
ACP^b^		600.88 ± 53.40^a^	698.15 ± 14.23^b^	780.46 ± 23.98^c^	784.08 ± 13.75^c^	774.52 ± 13.8^c^	774.81 ± 48.41^c^
C3^c^		35.20 ± 1.47^a^	44.07 ± 3.83^b^	51.63 ± 1.67^c^	60.03 ± 3.33^d^	52.85 ± 2.79^c^	52.40 ± 4.04^c^
C4^d^		7.40 ± 0.59^a^	8.89 ± 0.55^b^	11.10 ± 0.70^c^	11.03 ± 0.66^c^	11.17 ± 0.51^c^	11.08 ± 0.85^c^
Spleen							
LZ		206.55 ± 13.86^a^	234.67 ± 9.03^b^	258.00 ± 7.53^c^	296.18 ± 19.54^d^	291.87 ± 17.91^d^	293.75 ± 17.03^d^
ACP		676.68 ± 43.18^a^	786.99 ± 24.59^b^	861.49 ± 37.81^c^	975.62 ± 45.26^d^	967.75 ± 29.3^c d^	980.37 ± 69.06^d^
C3		75.96 ± 3.27^a^	92.57 ± 4.72^b^	116.89 ± 6.80^c^	121.94 ± 8.07^c^	119.20 ± 3.11^c^	95.70 ± 9.56^b^
C4		10.18 ± 0.94^a^	13.06 ± 0.93^b^	12.97 ± 0.30^b^	13.05 ± 1.07^b^	12.41 ± 0.48^b^	12.53 ± 0.39^b^
Regression							
Y _LZ in the head kidney_ = -0.3367*x* ^2^ + 11.1144*x* + 99.5644						*R*² = 0.8739	*P* < 0.05
Y_ACP in head kidney_ = 26.6600*x* + 493.9000; *Y*_max_ = 778.47						*R* ² = 0.9991	*P* < 0.01
Y_C3 in head kidney_ = - 0.1542*x* ^2^ + 4.9308*x* + 17.1462;						*R* ² = 0.9154	*P* < 0.05
Y_C4 in head kidney_ = 0.5430*x* + 5.0690; *Y*_max_ = 11.09						*R* ² = 0.9702	*P* < 0.05
Y_LZ in spleen_ = 8.7528*x* + 168.9600; *Y*_max_ = 293.93						*R* ² = 0.9890	*P* < 0.01
Y_ACP in spleen_ = 29.1480*x* + 433.7157; *Y*_max_ = 974.58						*R* ² = 0.9969	*P* < 0.01
Y _C3 in the spleen_ = -0.4440*x* ^2^ + 12.8638*x* + 27.8609						*R* ² = 0.9375	*P* < 0.05

Different superscript letters in the same column are significantly different (P < 0.05).

^a^LZ, lysozyme; ^b^ACP, acid phosphatase; ^c^C3, complement 3; ^d^C4, complement 4.

Values are means representing six fish per group.

### Antimicrobial Peptide and Inflammatory Cytokines in the HK and SP of Juveniles *In Vivo*

**Figure 3:**
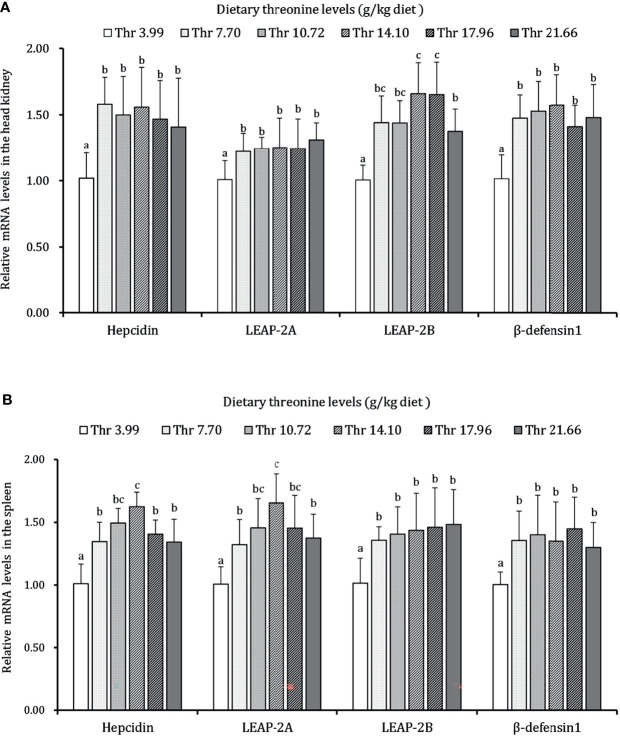
Relative mRNA expression of antimicrobial peptides in the head kidney **(A)** and spleen **(B)** of juvenile grass carp (*Ctenopharyngodon idella*) fed diets containing graded levels of Thr for 8 weeks. LEAP-2A, liver expressed antimicrobial peptide 2A; LEAP-2B, liver expressed antimicrobial peptide 2B; β-defensin1, beta-defensin 1. Data represent the means of six fish in each group. Error bars indicate SD. Values having different letters are significantly different (*P* < 0.05).

**Figure 4:**
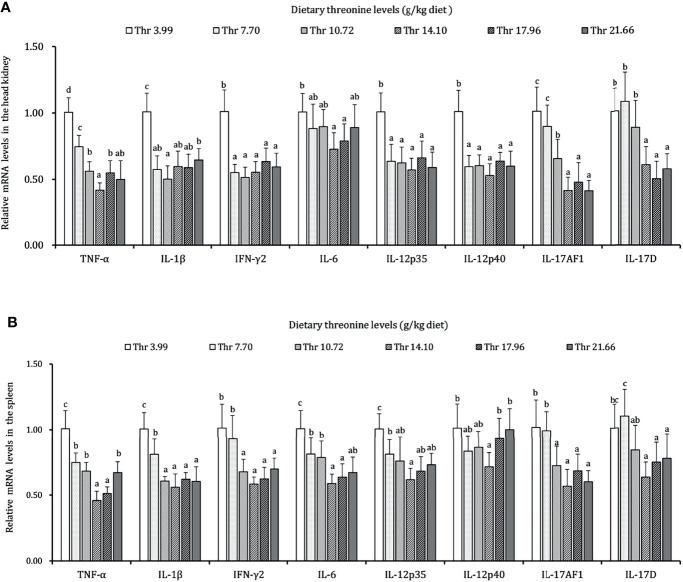
Relative mRNA expression of pro-inflammatory cytokines in the head kidney **(A)** and spleen **(B)** of juvenile grass carp (*Ctenopharyngodon idella*) fed diets containing graded levels of Thr for 8 weeks. TNF-α, tumor necrosis factor α; IFN-γ2, interferon gamma 2; IL, interleukin. Data represent the means of six fish in each group. Error bars indicate SD. Values having different letters are significantly different (*P* < 0.05).

**Figure 5:**
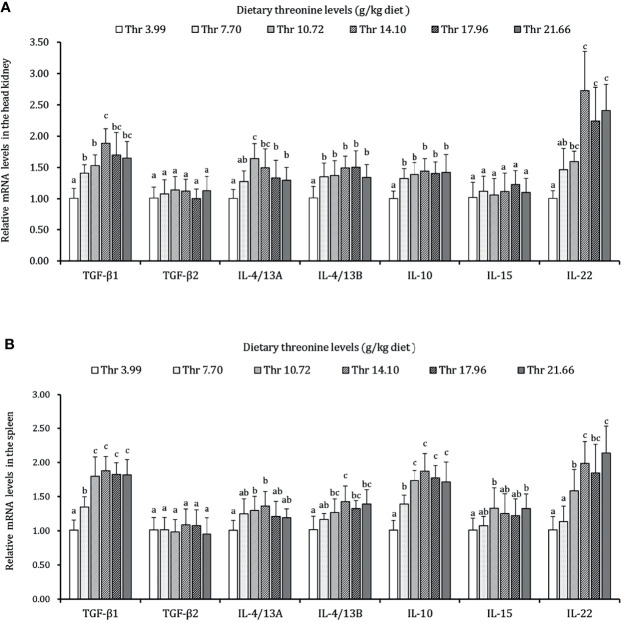
Relative mRNA expression of anti-inflammatory cytokines in the head kidney **(A)** and spleen **(B)** of juvenile grass carp (*Ctenopharyngodon idella*) fed diets containing graded levels of Thr for 8 weeks. TGF-β1, transforming growth factor β 1; TGF-β2, transforming growth factor β 2; IL, interleukin. Data represent the means of six fish in each group. Error bars indicate SD. Values having different letters are significantly different (*P* < 0.05).

As presented in **Figures 3**–**5**, dietary Thr has influenced the mRNA transcript abundances of anti-microbial peptides and inflammatory cytokines in the HK and SP of juveniles *in vivo*. In the HK, the LEAP-2B, IL-4/13A, and TGF-β1 mRNA abundances were increased as dietary Thr increased up to 14.10 g/kg, and then all went down. The hepcidin, LEAP-2A, β-defensin1, IL-4/13B, IL-10, and IL-22 mRNA expression levels displayed the lowest values in juveniles fed a Thr deficient-diet. The mRNA abundances of TNF-α, IL-6, and IL-1β showed a descending trends as dietary Thr was increased until the maximum level was obtained in juveniles fed 14.10 g/kg Thr diet and was then upregulated. Juveniles fed a Thr-insufficient diet showed maximum mRNA transcript levels of IFN-γ2, IL-12p35, IL-12p40, IL-17AF1, and IL-17D, respectively (*P* < 0.05) but, notably, not alteration in TGF-β2 and IL-15 (*P* > 0.05). In the SP, as dietary Thr increased, the mRNA expression levels of hepcidin, LEAP-2A, IL-4/13A, and IL-4/13B were upregulated, and the peak was acquired when juveniles were fed 14.10-g/kg Thr diet and was subsequently downregulated. As the incremental dietary Thr level increased from 3.99 to 10.72 g/kg, the mRNA transcript abundances of TGF-β1 and IL-10 increased significantly (*P* < 0.05) with no further upregulation by continuous Thr addition (*P* > 0.05). The minimum mRNA expressions of LEAP-2B and β-defensin1 were obtained in juveniles fed 3.99 g/kg Thr diet (*P* < 0.05). Inversely, as the dietary Thr levels increased, the mRNA expressions of TNF-α, IL-6, IL-12p35, IL-12p40, IL-17AF1, and IL-17D were reduced gradually to the lowest level when the dietary Thr level reached 14.10-g/kg, and then all increased. Compared with insufficient Thr, dietary Thr level above 10.72 g/kg enhanced the mRNA transcript levels of IFN-γ2 and IL-1β significantly (*P* < 0.05) and then plateaued (*P* > 0.05). A slightly increased IL-15 mRNA abundance was found in juveniles fed 10.72-g/kg Thr diet. Interestingly, the TGF-β2 mRNA abundance showed no difference in response to dietary Thr (*P* > 0.05).

### Adaptive Immune Response of Immunoglobulin Isoforms in the HK and SP of Juveniles *In Vivo*

**Figure 6:**
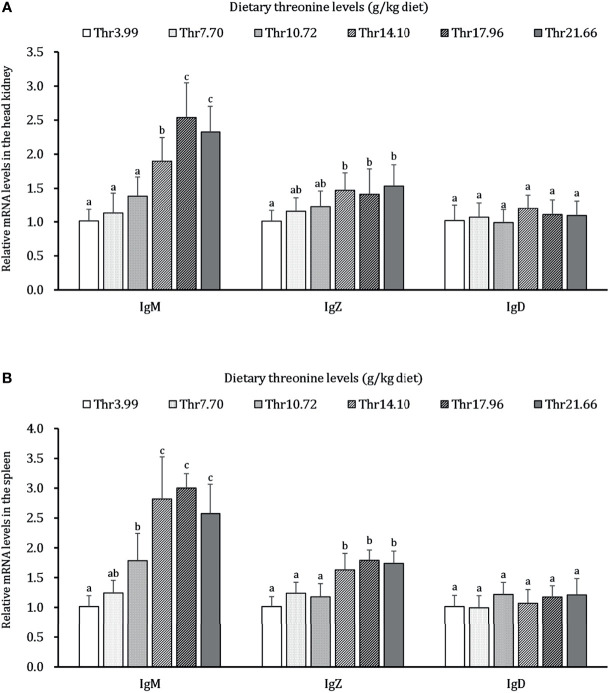
Relative mRNA expression of immunoglobulin isoforms in the head kidney **(A)** and spleen **(B)** of juvenile grass carp (*Ctenopharyngodon idella*) fed diets containing graded levels of Thr for 8 weeks. IgM, immunoglobulin M; IgZ, immunoglobulin Z; IgD, immunoglobulin; D, Data represent the means of six fish in each group. Error bars indicate SD. Values having different letters are significantly different (*P* < 0.05).

As presented in **Figure 6**, dietary Thr has a different regulation for the mRNA transcripts of immunoglobulin isoforms in the HK and SP of juveniles *in vivo*. In the HK, the significantly (*P* < 0.05) upregulated mRNA abundances of IgM were found with incremental levels of Thr and reached 2.5-fold changes when the fish was supplemented with 17.96-g/kg Thr diet. Compared with the Thr-insufficient-diet group (3.99g/kg), juveniles fed Thr above 14.10 g/kg showed 1.5-fold upregulation of IgZ mRNA expression unit. In the SP, the mRNA level of IgM was significantly (*P* < 0.05) upregulated and achieved the highest relative value (2.7-fold change) when dietary Thr was 14.10 g/kg and then plateaued (*P* > 0.05). Generally increased abundances of IgZ were affected with a maximum of 1.8-fold changes by 14.10 g/kg Thr. Dietary Thr did not alter the mRNA abundances of IgD in the HK and SP (*P* > 0.05).

### Immune-Related Signaling Molecules in the HK and SP of Juveniles *In Vivo*

**Figure 7:**
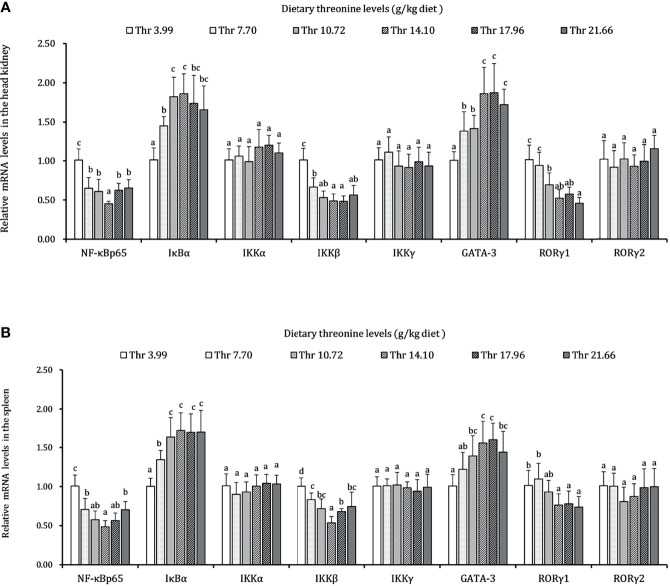
Relative mRNA expression of inflammation-related signaling molecules in the head kidney **(A)** and spleen **(B)** of juvenile grass carp (*Ctenopharyngodon idella*) fed diets containing graded levels of Thr for 8 weeks. NF-κB p65, nuclear factor kappa B p65; IκBα, inhibitor of κB α; IKKα, IκB kinase α; IKKβ, IκB kinase β; IKKγ, IκB kinase γ; GATA-3, GATA binding protein 3; RORγ, RAR-related orphan receptor gamma. Data represent the means of six fish in each group. Error bars indicate SD. Values having different letters are significantly different (*P* < 0.05).

The regulations of Thr on the mRNA expression level of immune-related NF-κB signaling (p65), inhibitor protein κBα (IκBα), and IκB kinases (α, β, and γ subunits) are displayed in **Figure 7**. In the HK, the mRNA transcript abundances of NF-κB p65 and IKKβ showed downregulation responses to the incremental Thr level from 3.99 to 14.10 g/kg and then displayed the ascending tendency gradually. An enhanced mRNA level was found in IκBα, and it reached a peak as the incremental Thr level increased from 3.99 to 14.10 g/kg and then decreased gradually. The GATA-3 and RORγ1 mRNA levels were upregulated gradually by dietary Thr and obtained the highest level when juveniles were fed 14.10 g/kg diet (*P* < 0.05) and then plateaued (*P* > 0.05). There were no dramatic differences in IKK (α and γ) mRNA level responses to dietary Thr (*P* > 0.05). In the SP, the mRNA expression of NF-κB p65 and IKKβ showed slightly declining trends until the dietary Thr level was up to 14.10 g/kg, and it gradually increased thereafter. The GATA-3 and RORγ1 transcript abundances were upregulated as the Thr levels increased from 3.99 to 14.10 g/kg diet and then dropped off. Significant (*P* < 0.05) IκBα mRNA abundance was upregulated with the achieved highest value in juveniles fed dietary Thr at 10.72 g/kg, and the relative stable expression was kept thereafter (*P* > 0.05). Unexpectedly, the mRNA expression levels of IKKα, IKKγ, and RORγ2 were unchanged dramatically (*P* > 0.05) by dietary Thr.

### Macrophage Activators, Cytokines, and Signaling Regulators in the HK of Juveniles *In Vitro*

**Figure 8:**
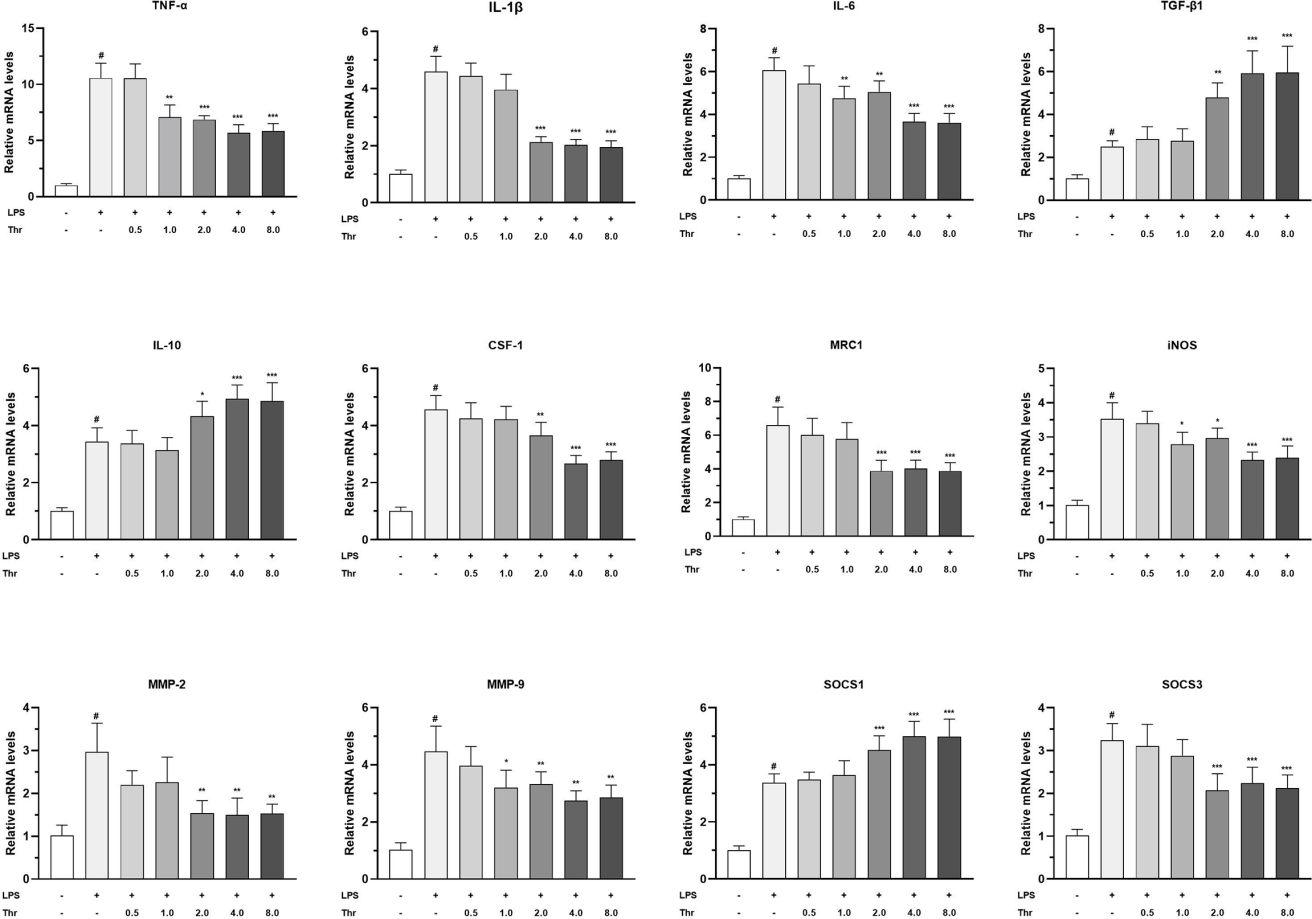
Effect of graded levels of L-Thr (0, 0.5, 1.0, 2.0, 4.0, and 8.0 mM) on the mRNA expression of inflammatory cytokines and related signaling molecules in the macrophages of grass carp (*Ctenopharyngodon idella*) head kidney under lipopolysaccharide (LPS) induction. TNF-α, tumor necrosis factor α; IL, interleukin; TGF-β1, transforming growth factor β 1; CSF-1, colony-stimulating factor 1; MRC1, mannose receptor C-type 1; iNOS, inducible nitric oxide synthase; SOCS1, suppressor of cytokine signaling 1; SOCS3, suppressor of cytokine signaling 3; MMP, matrix metalloproteinase; Thr, threonine. Data represent the means of six replicates in each group. Error bars indicate SD. Columns marked with “#” represent significant difference compared with the control group “LPS- and Thr–”(*P* < 0.05), and those marked with “*, **, ***” represent significant difference compared with “LPS+ and Thr–”group (P<0.05, 0.01, 0.01).

The targeted effects of macrophage activators, inflammatory cytokines, and signal regulators by dietary Thr in the HK macrophages are presented in **Figure 8**. In macrophages of the head kidney, compared with the control group, LPS upregulated the mRNA transcript abundances of TNF-α, IL-1β, IL-6, IL-10, TGF-β1, CSF1, MRC1, iNOS, MMP-2, MMP-9, SOCS1, and SOCS3 significantly (*P* < 0.05). Compared with the LPS-simulated group, 1.0 mM L-Thr supplementation downregulated the TNF-α, iNOS, and IL-6 mRNA expression abundances significantly (*P* < 0.01). The medium supplemented with 2.0 mM L-Thr decreased the mRNA levels of IL-1β, CSF-1, MMP-2, MMP-9, MRC1, and SOCS3 and increased the TGF-β1, IL-10, and SOCS1 expression abundances significantly (*P* < 0.01).

### Protein Levels in the HK and SP of Juveniles *In Vivo* and *In Vitro*

**Figure 9:**
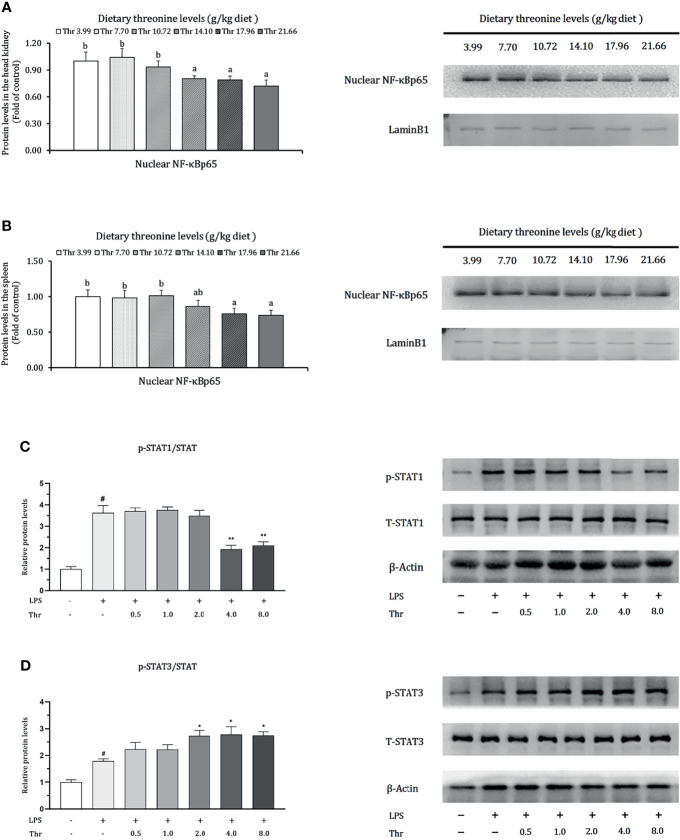
Protein levels of NF-κB p65 in the head kidney **(A)**, spleen **(B)**, STAT1 **(C)**, and STAT3 **(D)** in the macrophages of head kidney of grass carp (*Ctenopharyngodon idella*). NF-κB p65, nuclear factor kappa B p65; T-STAT, total protein of signal transducer and activator of transcription; p-STAT1, phospho-STAT1(Tyr701); p-STAT3, phospho-STAT3 (Tyr705); LPS, lipopolysaccharide; Thr, threonine. Data represent triplicates of each group. Error bars indicate SD. Values having different letters are significantly different in **(A, B)** (*P* < 0.05). Columns marked with “#” represent significant difference compared with the control group “LPS- and Thr-”(*P* < 0.05), and those marked with “*,**” represent significant difference compared with “LPS+ and Thr –” group (*P* < 0.05, 0.01) in **(C, D)**.

Data regarding the modulation of dietary Thr on the protein expression of NF-κBp65 in the HK and SP and in STAT1 and STAT3 in macrophages of the head kidney are presented in **Figure 9**. In the HK, compared with Thr deficiency, juvenile fed dietary Thr at a level above 14.10 g/kg obtained lower protein expression abundances of nuclear NF-κBp65 (*P* < 0.05). In the SP, as the incremental Thr level was increased from 3.99 to 14.10 g/kg, the protein expression of nuclear NF-κBp65 declined gradually and plateaued (*P* > 0.05) thereafter. In the macrophages of HK, compared to the control group (without LPS and L-Thr addition), the protein expression levels of phospho-STAT1 (Tyr701) and phospho-STAT3 (Tyr705) were all enhanced significantly (*P* < 0.05) after LPS stimulation for 6 h. Compared with the LPS-stimulated group, supplementation of above 4 mM L-Thr in the medium downregulated the protein relative expression units of phospho-STAT1 (Tyr701), and 2 mM L-Thr could upregulate the expression abundances of hosphor-STAT3 (Tyr705) significantly (*P* < 0.05).

## Discussion

Nutritional deficiency has been focused on the alteration of immunological function by experimental and clinical investigation for many years in animals (25). Compared with the mammalians, amino acids are the more principal nutrients in teleost not only for body composition but also as the main donors for energy. Teleost HK and SP integrated the recruitment center of immunocytes for self-renewal in a diffused lymphatic system (gill, intestine, skin, *etc.*). To date, few evidence were addressed on the effects of amino acids on immune responses *in vivo*, and what is more, there was still a lack of *in vitro* study for comparison. Thus, this study, for the first time, explores immunoregulation by amino acid with the example of Thr *in vivo* and *in vitro* study of the teleost grass carp (*Ctenopharyngodon idella*).

### Thr Improved the Immune Status and Mediated the Inflammation Responses *In Vivo*

Like mammals, teleost have been fundamentally concluded to have the same features during immune activation, such as antigen presentation, phagocytosis, and T lymphocyte response (71, 72). The highly expressed MHC-II commonly served as open access to the link from APCs to dendritic cells and B and T lymphocytes (73). In this study, our data displayed that, compared with the Thr-insufficient group (3.99 g/kg), the optimal dietary Thr level (14.10 g/kg) downregulated the mRNA relative expressions of MHC-IIα and MHC-IIβ in the HK and SP of juvenile grass carp after having been challenged by *A. hydrophila*, implying that dietary Thr might attenuate the immune reaction by the reduced activation of immunocytes after receiving stimulation from antigens. As the crucial achieving regulators in T cells, CD4 and CD8 were primarily the surface molecules that characterize differentiation and maturation (74, 75). However, in our present study, the supplemented optimal dietary Thr increased the CD4 gene expression in the HK and SP, demonstrating that the optimal dietary Thr addition could promote the motivation of CD4^+^ immunocytes in fish. It was reported that the alternative activation of CD4^+^ T cells could be circumvented by MHC-II in mice (76) despite the common sense that MHC-II-expressed immunocytes tend to mobilize the invasion signals to CD4 expression T cells. Accumulating data confirmed that Tim-4-expressed APCs could promote T cell survival and division in mice (77, 78). Structurally, there exists the immunoglobulin- and mucin-domain-containing unit in Tim-4 (79). Performing the specific role in barrier function maintenance, the synthesis of mucin and immunoglobulins was limited by Thr in animals (80–82). Thus, the discrepancy expression patterns of MHC-II and CD4 might be independent modulation by Thr. Interestingly, dietary Thr did not influence the CD8 gene expression in the HK and SP, which might be related to GATA-3. As a conventional transcription factor, GATA-3 was required for the development and maturation of CD4, but not CD8, thymocytes in mice (83). Baraut et al. (84) reported that GATA-3 could be upregulated by TGF-β in human T cells. In our study, we have found that the dietary Thr promoted the TGF-β1 and GATA-3 gene transcripts in the HK and SP, thus supporting our hypothesis. Besides this, CD8 was confirmed to stabilize MHC-I docking with T-cell antigen receptors by affinity measurement (85). In the ileum of piglets, dietary Thr supplementation downregulated the MHC-I mRNA abundance (42). This indicated that the unchanged CD8 mRNA level by Thr might be an unnecessary transcript appeal to stabilize the decline of MHC-I mRNA expressions in the HK and SP of teleost fish. Considering that little evidence addressed the effect of Thr on immunocyte activation, it still needs further investigation.

Once awakened by antigens in an immunologic process, innate immune factors produced by immunocytes, like LZ and C3 derived by macrophages, ACP released by leukocytes simultaneously executed the defense role by breaking down the foreign antigens in the immune organs of fish (86). In our study, compared with the Thr-insufficient-diet group, the optimal Thr level enhanced the enzyme activity of LZ and ACP, complement contents (C3 and C4), and gene expression of antimicrobial compounds hepcidin, LEAP-2A, LEAP-2B, and β-defensin1 in the HK and SP of juvenile grass carp after the challenge with *A. hydrophila*. The above-mentioned results manifested that incremental dietary Thr could enhance the immune defense in the HK and SP of fish. Similar phenomena were observed on arginine in juvenile Jian carp (*Cyprinus carpio* var Jian) (87) and lysine in grown-up grass carp (88). Research confirmed that transcription factor SP1 targeted the lysozyme expression in A549 cells (89) and could motivate the complement system with complement 4b-blinding upregulation in HepG2 cells (90). Copland et al. have found that IGF-1 could upregulate the SP1 expression in human fetal muscle (91). Reportedly, amino acids magnified the metabolic and reproductive function partly reflected in the increased circulation of IGF-1 in animals (92, 93). From observations, it was forecast that the increased production of immune components LZ and complements by dietary Thr might be ascribed to the upregulation of SP1 by the efforts of IGF-1 in fish. However, further investigation is needed to support this idea.

Furthermore, the secreted cytokines were triggered not only for immunocyte stimulation but also immune homeostasis in teleost (94). Generally, the boosted inflammation was established as upregulation of pro-inflammatory cytokines (*e*.*g*., TNF-α, IL-6, and IL-12) and the suppression of anti-inflammation (*e*.*g*., IL-10 and TGF-β) in animals (95, 96). In our study, compared to Thr insufficiency, the optimal Thr level in the diet decreased the transcript abundances of pro-inflammation cytokines TNF-α, IL-1β, IFN-γ2, IL-6, IL-12p35, and IL-12p40 and increased the anti-inflammation-related IL-10, TGF-β1, IL-4/13A, and IL-4/13B in the HK and SP of juvenile grass carp. The decreased expression of RORγ1 regulator by dietary Thr were also coordinated with the downregulated IL-17AF1 and IL-17D, which implied a possible suppression of T helper 17 cell motivation by Thr during the inflammatory process. The above-mentioned data indicated that dietary Thr supplementation could mediate an inflammation process in the HK and SP of fish partly by attenuating the pro-inflammation and strengthening the anti-inflammation effects. Evidence of agreement was obtained as a similar trend was affected by other amino acids like lysine in grown-up grass carp (88). Interestingly, TGF-β1 (not TGF-β2) mRNA expression was increased by dietary Thr supplementation in the HK and SP of juvenile grass carp. This phenomenon might be concerned with methionine altering insulin-EGF signaling. Sarwar et al. (97) confirmed that dietary Thr could induce the accumulation of methionine levels in the plasma of rat. In mice, studies showed that methionine increased the insulin level, which could potentiate EGF signaling (98, 99). It was claimed that EGF could increase TGF-β1 (not β2) gene expression in mice (100). Hence, the possibility that dietary Thr upregulated the TGF-β1 (rather than TGF-β2) gene transcript levels might be partly caused by extending the methionine-targeted enhancement of insulin-EGF signaling in fish. However, this speculation needs further verification. Besides that, complicated inflammatory networks could be compatible between the traditional NF-κB signaling target enlargement of pro-inflammation production and GATA-3-mediated anti-inflammation effects (101, 102). It was documented that the inactivation of IKK complexes (α, β, γ) repressed IκBα degradation and then inhibited the activation of NF-κB p65 (103). In our study, compared with Thr deficiency, the optimal dietary Thr level induced the downregulation of NF-κB p65 and IKKβ (not α and γ) in the gene transcript levels and nuclear NF-κB p65 protein levels and the upregulation of GATA-3 mRNA expression in the HK and SP of juvenile grass carp. The correlation index (**Supplementary Table S2**) proved that those gene transcripts of pro-inflammatory cytokines (TNF-α, IL-1β, IFN-γ2, IL-6, and IL-12p35) were positively related to NF-κB p65, and anti-inflammation-related cytokines (TGF-β, IL-10, and IL-4) were forwardly correlated with GATA-3. Furthermore, the gene transcript levels of IκBα showed adverse relevance with IKKβ and NF-κB p65, suggesting that optimal dietary Thr supplementation modulated the inflammatory response partly associated with reducing the NF-κBp65 axis and amplifying the GATA-3 signaling in fish. Interestingly, the optimal dietary Thr level decreased the IKKβ (not α and γ) gene expression in the HK and SP of juvenile grass carp, which might be relate to IFN-γ2. In Hela cells, IFN-γ2 could induce the decrease of mRNA transcript abundance of N-myc downstream-regulated gene 1 (NDRG1) (104). It was demonstrated that the repression of NDRG1 could lead to an upregulation of IKKβ but ignored the IKKα and IKKγ expression in mice (105). In our study, the supplemented dietary Thr downregulated the IFN-γ mRNA abundances in the HK and SP of juvenile grass carp. Hence, we speculated that the downregulation of IKKβ (not α and γ) by dietary Thr might be partly associated with the declined IFN-γ mRNA, thus resulting in NDRG1 upregulating the expression in the HK and SP of juvenile grass carp. However, the underlying mechanism requires more verification. Thus, to interpret the mechanism on whether the potential immunoregulation by Thr was corroborated in immunocytes, we next isolated the macrophages in the head kidney to probe the study *in vitro*.

### Thr Modulated the Inflammation Homeostasis in HK Macrophages of Grass Carp *In Vitro*

Macrophages executed the basic role of phagocytosis and immune system motivation (106). The activated macrophages could be mainly characterized as canonical M1 and M2 types, which were in charge of the pro- and anti-inflammation responses, respectively. The immune metabolic reprogramming of biomarkers in fish macrophages has already been suggested to be similar with that of mammalians (107). In this study, 40 ug/ml LPS was employed as having the immunostimulatory properties to induce grass carp HK macrophages for 6 h with the upregulated gene expression of macrophage activators (iNOS, CSF-1, and MRC-1) and inflammation cytokines (TNF-α, IL-1β, and IL-6) with at least 3 times fold change, which were subsequently reduced by 2 mM L-Thr supplementation. It was reported that CSF-1 potentially drove the differentiation and polarization during the M1 type macrophage remodeling in mice and was involved in pro-inflammation boost with TNF-α, iNOS, IL-1β, and matrix metalloproteinases (108–110). We also found that the release of MMP-2 and MMP-9, potentially by canonical macrophage activation, was downregulated by treatment with 2 mM Thr. Thus, grass carp HK macrophages for M1 type deactivation by Thr could be due to pro-inflammatory remission. Moreover, IL-10 derived from M2 type macrophages could enhance the anti-inflammation effect and, conversely, serve as a reinforcement stimulation for feedback (111). In this study, compared with LPS induction without L-Thr, the further increase in IL-10 and TGF-β1 mRNA levels by 2 mM L-Thr addition suggested the promotion of an anti-inflammatory effect which can be partly ascribed to the benefit of M2 type macrophages in HK of fish. In depth, cytokine intervention on the tendency of macrophage phenotype depended on STATs coupling with SOCS modulation (112). Evidences were given to show that the suppression of SOCS1 could elicit STAT1 signaling for polarizing the M1 type macrophages, and retardation of SOCS3 could induce STAT3 activation for M2 type proceeding to anti-inflammation (113, 114). Our data showed that LPS induced remarkable increases in the protein levels of STAT1 and STAT3, and the treatment with 4 mM L-Thr caused reverse downregulation in STAT1 and continuous upregulation in STAT3. Correspondingly, 2 mM of L-Thr upregulated the SOCS1 and downregulated the SOCS3 gene expression in HK macrophages. These data suggested that L-Thr alleviated the inflammation by maintaining the HK macrophage homeostasis with the efforts of SOCS1/STAT1 signaling restriction and SOCS3/STAT3 enlargement. To date, limited studies addressed the macrophage’s substantial fate by amino acids but is typically observed in arginine, of which derived iNOS and the involvement by arginase 1 were recognized as the vast changes during the early stage of macrophage polarization (115). Except for the corroborative donors of Thr during immune responses by B lymphocyte stimulation which directly affect immunoglobulin production, our study primarily gives the first view of understanding Thr’s contribution to macrophage function in teleost fish.

### Comparison of the Dosage Effect of Thr on Immunomodulation

Obviously, compared with Thr deficiency *in vivo*, the optimal Thr level could modulate the immune response, resulting in pro-inflammation suppression and improvement of anti-inflammation. However, changes by excess Thr, in most of the cases the immune index including immunocyte biomarker CD molecules, enzymes LZ and C3, antimicrobial peptides LEAP2 and β-defensin, as well as cytokines (IL-1β, IL-6, and IL-10), were not notable compared with the optimal dietary Thr group. However, compared with HK and SP as focus of this study, there exist functional segment differences donated to the immune status by dietary Thr during local immune response (*e*.*g*., intestine) as previously reported (56), which suggests that immunoregulation is heterogeneous in different organs of fish. Based on the immune-related index (LZ activity in the HK and C3 content in the SP), the optimal dietary Thr levels for immune improvement in juvenile grass carp are estimated to be 15.70 g/kg diet (4.85 g/100 g protein) and 14.49 g/kg diet (4.47 g/100 g protein), respectively, which are close to or slightly higher than that on the growth requirement with 14.53 g/kg diet (4.48 g/100 g protein) (56), suggesting that a little more Thr is required for enhancing the immune status of teleost fish.

## Conclusion

**Figure 10:**
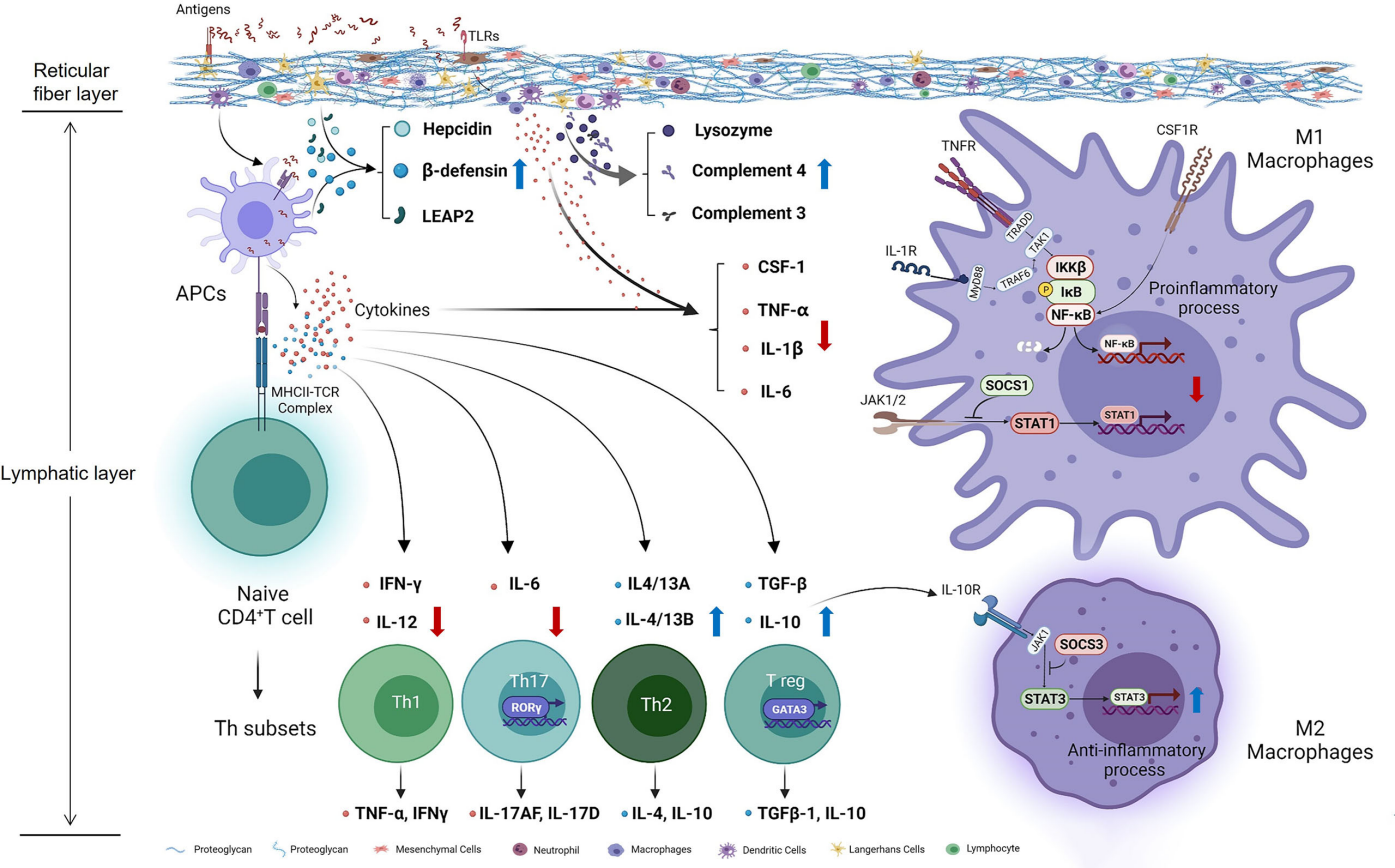
Potential action pathways of Thr on immune regulation in the head kidney of teleost fish. Arrows marked with blue and red colors represent the positive and negative effects by threonine, respectively.

Taken together, our study preliminary investigated the effects of Thr on immunomodulation *in vivo* and *in vitro* of fish (**Figure 10**). Our data showed that, compared with dietary Thr deficiency, the optimal Thr supplementation modulated the immune response in the HK and SP with downregulation of the immunocyte biomarkers MHC-II and upregulation of CD4, increasing the activities of immune defense substances LZ and ACP, contents of C3 and C4, mRNA abundances of hepcidin, LEAP-2A, LEAP-2B, and β-defensin1, upregulating the mRNA expression of anti-inflammatory cytokines (except TGF-β2) associated with activated GATA-3, and downregulating the pro-inflammation-related cytokines as well as weakened iκB/NF-κBp65/IKKβ (not α and γ) signaling. L-Thr mitigated the inflammation *in vitro* by downregulating the gene expression of macrophage activators (CSF-1, iNOS, and MRC-1) and cytokines (TNF-α, IL-1β, and IL-6) and upregulating IL-10 as well as impairing SOCS1/STAT1 and intensifying the SOCS3/STAT3 pathway in HK macrophages of fish. Additionally, based on the immune index (LZ activity in the HK and C3 content in the SP), the optimal Thr levels for immune enhancement in juvenile grass carp are estimated to be 15.70 g/kg diet (4.85 g/100 g protein) and 14.49 g/kg diet (4.47 g/100 g protein), respectively.

## Data Availability Statement

All the data supporting this study are available for authors without reservation as response to any request.

## Ethics Statement

The animal study was reviewed and approved by the Animal Care Advisory Committee in Sichuan Agricultural University

## Author Contributions

Y-WD conducted the study and laboratory analysis and wrote and revised the original draft. W-DJ performed the project administration, data proofreading, and draft revising. PW performed the conceptualization, methodology, and data curation. YL performed project administration. S-YK, LT, and W-NT provided supporting experimental facilities. LF performed the conceptualization, draft editing, funding acquisition, and supervision. X-QZ performed the conceptualization, project design, funding acquisition, and supervision. All authors contributed to the article and approved the submitted version.

## Funding

This research was financially supported by the National Key R&D Program of China (2019YFD0900200 and 2018YFD0900400), the National Natural Science Foundation of China for Outstanding Youth Science Foundation (31922086), the Young Top-Notch Talent Support Program, China Agriculture Research System of MOF and MARA (CARS-45), and Sichuan Science and Technology Program (2019YFN0036).

## Conflict of Interest

The authors declare that the research was conducted in the absence of any commercial or financial relationships that could be construed as a potential conflict of interest.

## Publisher’s Note

All claims expressed in this article are solely those of the authors and do not necessarily represent those of their affiliated organizations, or those of the publisher, the editors and the reviewers. Any product that may be evaluated in this article, or claim that may be made by its manufacturer, is not guaranteed or endorsed by the publisher.
