# Supplementary figures and images for: Novel Insight Into Nutritional Regulation in Enhancement of Immune Status and Mediation of Inflammation Dynamics Integrated Study In Vivo and In Vitro of Teleost Grass Carp (Ctenopharyngodon idella): Administration of Threonine
Source: Front Immunol. 2022 Mar 14;13:770969. doi: 10.3389/fimmu.2022.770969 (PMC8963965; doi:10.3389/fimmu.2022.770969)

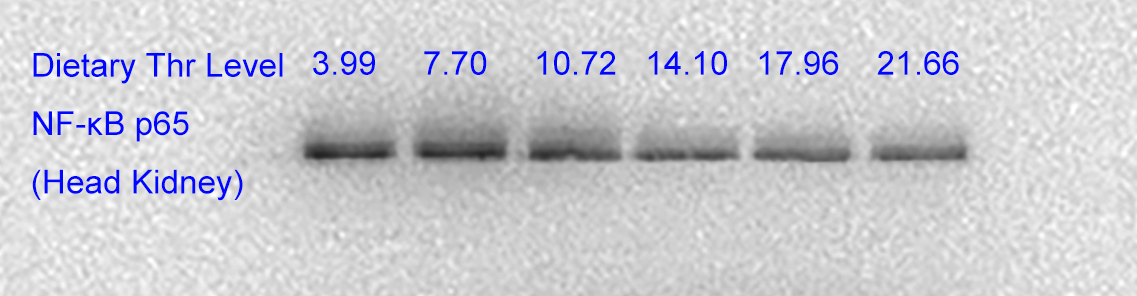

Supplement: Supplementary file 1 [file Image_1.tif]

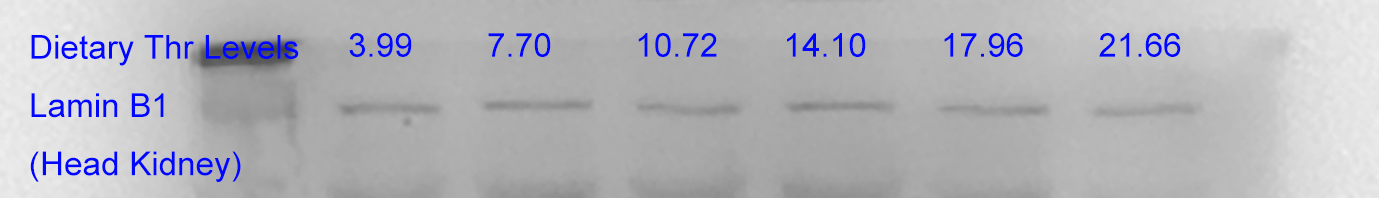

Supplement: Supplementary file 2 [file Image_2.tif]

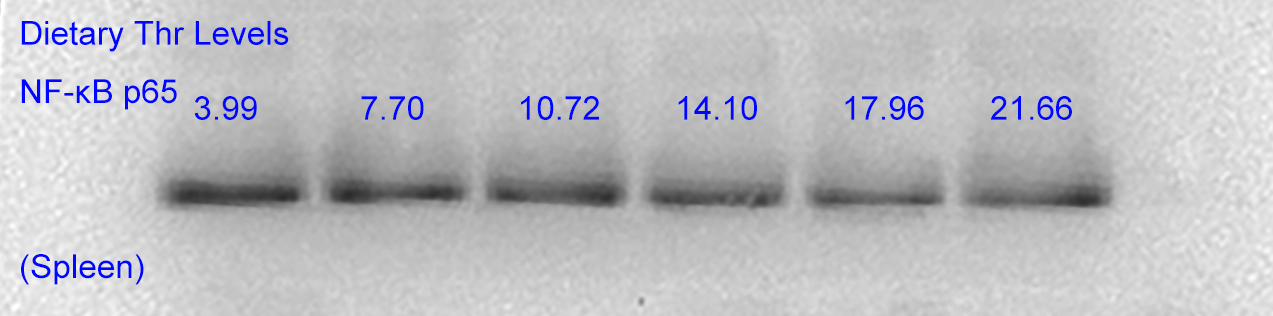

Supplement: Supplementary file 3 [file Image_3.tif]

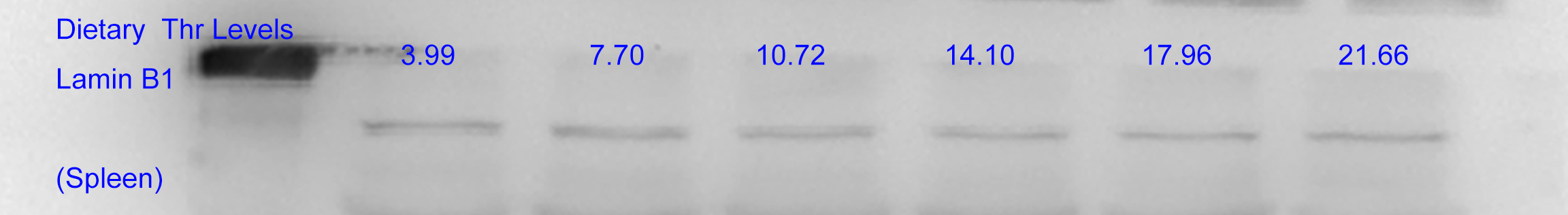

Supplement: Supplementary file 4 [file Image_4.tif]

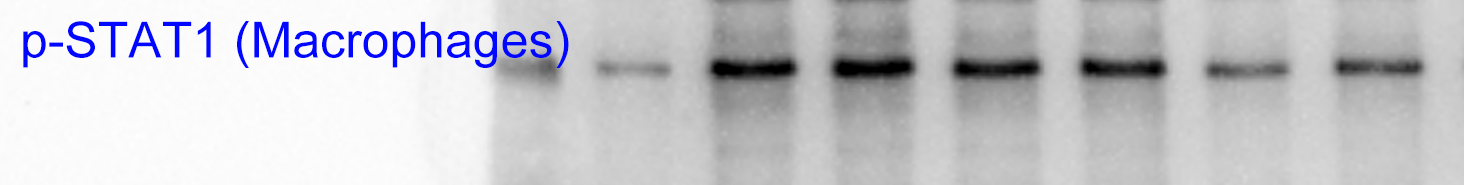

Supplement: Supplementary file 5 [file Image_5.tif]

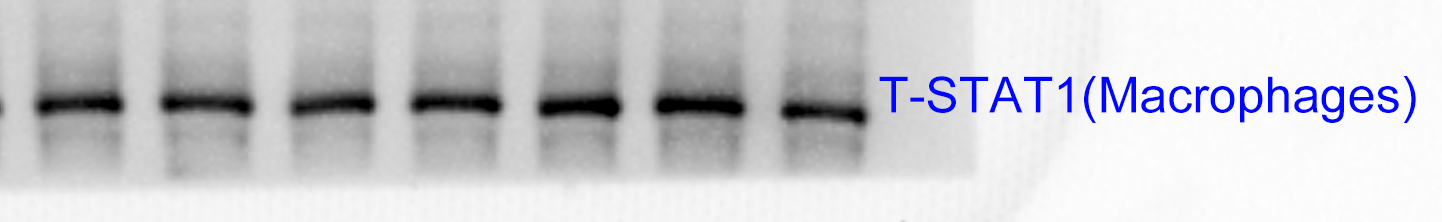

Supplement: Supplementary file 6 [file Image_6.tif]

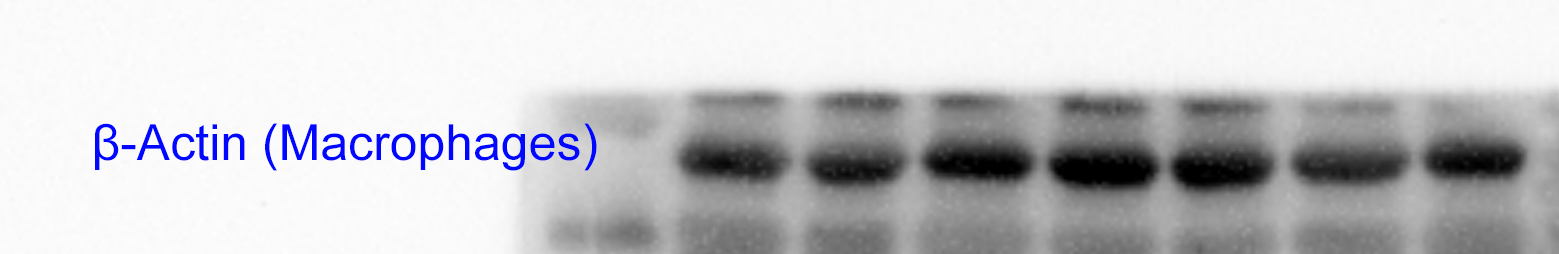

Supplement: Supplementary file 7 [file Image_7.tif]

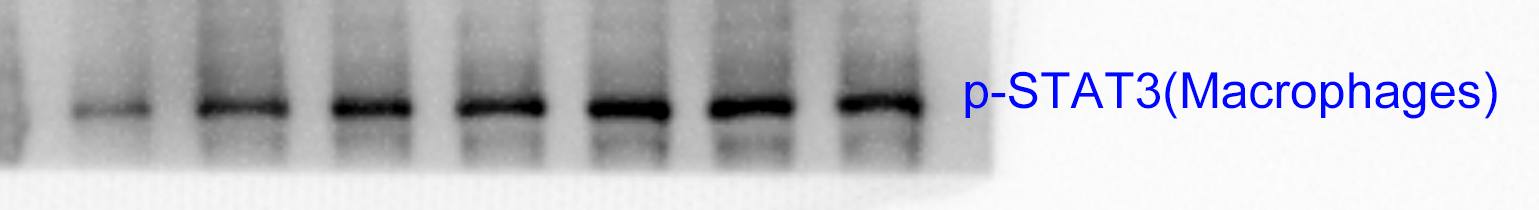

Supplement: Supplementary file 8 [file Image_8.tif]

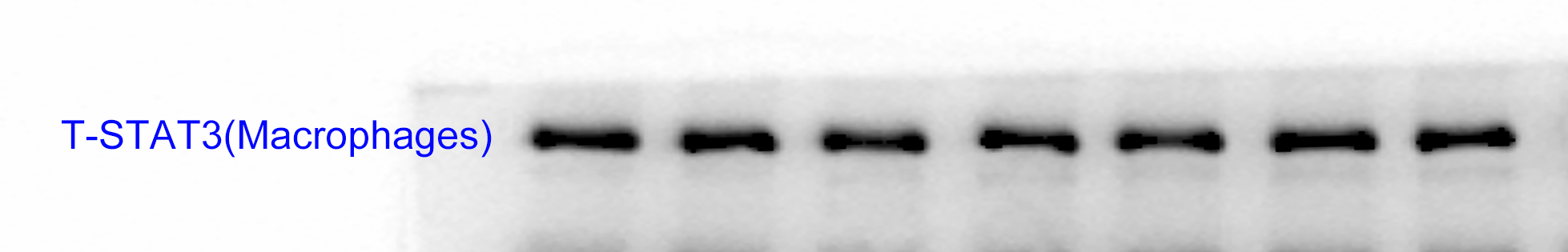

Supplement: Supplementary file 9 [file Image_9.tif]

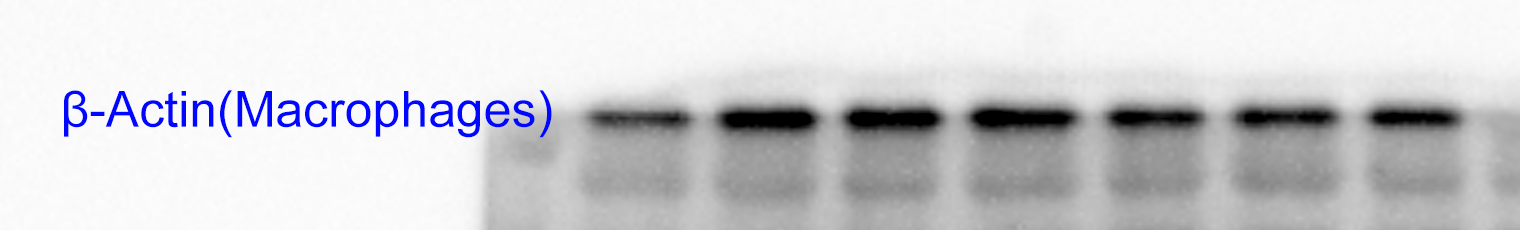

Supplement: Supplementary file 10 [file Image_10.tif]
