# Supplementary material for: Novel Insight Into Nutritional Regulation in Enhancement of Immune Status and Mediation of Inflammation Dynamics Integrated Study In Vivo and In Vitro of Teleost Grass Carp (Ctenopharyngodon idella): Administration of Threonine
Source: Front Immunol. 2022 Mar 14;13:770969. doi: 10.3389/fimmu.2022.770969 (PMC8963965; doi:10.3389/fimmu.2022.770969)
Supplement: Supplementary file 11 [file Table_1.docx]

**Supplement Table 1** Composition and nutrients content of basal diet

| Ingredients | g/kg | Nutrient contents ^a^ | g/kg |
| --- | --- | --- | --- |
| Fish meal | 50.4 | Crude protein | 324.0 |
| Casein | 30.0 | Crude lipid | 32.4 |
| Crystal amino acid mix ^b^ | 203.1 | ω-3 | 5.0 |
| Threonine premix ^c^ | 50.0 | ω-6 | 10.0 |
| Gelatin | 78.6 | Available phosphorus | 8.4 |
| Fish oil | 9.3 |  |  |
| Soyabean oil | 19.4 |  |  |
| α-starch | 290.0 |  |  |
| Corn starch | 150.5 |  |  |
| Ca (H_2_PO_4_)_2_ | 33.2 |  |  |
| Mineral premix ^d^ | 20.0 |  |  |
| Vitamin premix ^e^ | 10.0 |  |  |
| Choline chloride (60%) | 5.0 |  |  |
| Cellulose | 50.0 |  |  |
| Ethoxyquin (30%) | 0.5 |  |  |

^a^ Crude protein and crude lipid contents were measured value. Available phosphorus, n-3 and n-6 contents were calculated according to NRC (2011).

^b^ Crystal amino acid mix (g/kg): lysine, 11.42; methionine, 7.81; tryptophan, 2.88; arginine, 9.56; histidine, 7.86; leucine, 18.64; isoleucine, 10.30; phenylalanine, 10.22; tyrosine, 6.44; valine, 11.25; Cysteine, 2.87; glutamic acid, 57.50; glycine, 46.35, respectively.

^c^ Threonine premix was added to obtain graded levels of threonine, and the amount of glycine and corn starch was reduced to compensate. Per kilogram of threonine premix composition from diet 1 to 6 was as follows (g/kg): L-threonine 0.00, 71.40, 142.80, 214.20, 285.80, 357.20; glycine 222.80, 178.40, 133.80, 89.20, 44.60, 0.00 g and corn starch 777.20, 750.20, 723.40, 696.60, 669.60, 642.80g, respectively.

^d^ Per kilogram of mineral premix (g/kg): MnSO_4_.H_2_O (31.8% Mn), 1.8900; MgSO_4_⋅H_2_O (15.0% Mg), 200.0000; FeSO_4_.H_2_O (30.0% Fe), 24.5700; ZnSO_4_.H_2_O (34.5% Zn), 8.2500; CuSO_4_.5H_2_O (25.0% Cu), 0.9600; KI (76.9% I), 0.0668g; Na_2_SeO3 (44.7% Se), 0.0168. All ingredients were diluted with corn starch to 1 kg.

^e^ Per kilogram of vitamin premix (g kg^-1^): retinyl acetate (500,000IU/g), 2.10; cholecalciferol (500,000IU/g), 0.40; D, L-α-tocopherol acetate (50%), 12.58; menadione (22.9%), 0.83; cyanocobalamin (1%), 0.94; D-biotin (2%), 0.75; folic acid (95%), 0.42; thiamine nitrate (98%), 0.11; ascorhyl acetate (95%), 4.31; niacin (99%), 2.58; meso-inositol (98%), 19.39; calcium-D-pantothenate (98%), 2.56; riboflavin (80%), 0.63; pyridoxine hydrochloride (98%), 0.62. All ingredients were diluted with corn starch to 1 kg.
