# Supplementary material for: Novel Insight Into Nutritional Regulation in Enhancement of Immune Status and Mediation of Inflammation Dynamics Integrated Study In Vivo and In Vitro of Teleost Grass Carp (Ctenopharyngodon idella): Administration of Threonine
Source: Front Immunol. 2022 Mar 14;13:770969. doi: 10.3389/fimmu.2022.770969 (PMC8963965; doi:10.3389/fimmu.2022.770969)
Supplement: Supplementary file 12 [file Table_2.docx]

**Supplement Table 2** Correlation coefficient of parameters in the head kidney and spleen ^a^

| **Dependent parameters** | **Independent parameters** | **Head kidney** | | **Spleen** | |
| --- | --- | --- | --- | --- | --- |
|  |  | **Correlation coefficients** | ***P*** | **Correlation coefficients** | ***P*** |
| NF-κB p65 | TNF-α | + 0.926 | < 0.01 | + 0.955 | < 0.01 |
|  | IL-1β | + 0.878 | < 0.05 | + 0.925 | < 0.01 |
|  | IFN-γ2 | + 0.913 | < 0.05 | + 0.896 | < 0.05 |
|  | IL-6 | + 0.893 | < 0.05 | + 0.888 | < 0.05 |
|  | IL-12p35 | + 0.947 | < 0.01 | + 0.959 | < 0.01 |
|  | IL-12p40 | + 0.965 | < 0.01 | + 0.696 | = 0.12 |
| IκBα | IKKβ | - 0.976 | < 0.01 | - 0.894 | < 0.05 |
| GATA3 | TGF-β1 | + 0.960 | < 0.01 | + 0.950 | < 0.01 |
|  | IL-4/13A | +0.635 | = 0.23 | + 0.710 | = 0.11 |
|  | IL-4/13B | + 0.908 | < 0.05 | + 0.927 | < 0.05 |
|  | IL-10 | + 0.880 | < 0.05 | + 0.959 | < 0.01 |

^a^ NF-κB p65, Nuclear factor kappa B p65; TNF-α, Tumor Necrosis Factor α; IL, Interleukin; IFN-γ2, Interferon Gamma 2; IκBα, Inhibitor of κB α; IKKβ, IκB kinase β; TGF-β1, Transforming Growth Factor β 1;
